# Causes of Death in Congenitally Corrected Transposition of Great Arteries

**DOI:** 10.1016/j.jacadv.2024.101036

**Published:** 2024-06-11

**Authors:** Nabil Dib, Jean-Marc Sellal, Matilde Karakachoff, Ismail Bouhout, Vincent Chauvette, Nancy Poirier, Christopher J. McLeod, Lynne E. Nield, Paul Khairy, Alban-Elouen Baruteau

**Affiliations:** a Montreal Heart Institute, Université de Montréal, Montréal, Québec, Canada; b CHRU-Nancy, Université de Lorraine, Nancy, France; c Clinique des Données, CHU Nantes, INSERM, CIC 1413, Nantes, France; d Mayo Clinic, Rochester, Minnesota, USA; e Labatt Family Heart Centre, The Hospital for Sick Children, University of Toronto, Toronto, Ontario, Canada; f CHU Nantes, FHU PRECICARE, Nantes Université, Nantes, France; g CHU Nantes, INSERM, CIC FEA 1413, Nantes Université, Nantes, France; h CHU Nantes, CNRS, INSERM, l’institut du thorax, Nantes Université, Nantes, France; i INRAE, UMR 1280, PhAN, Nantes Université, Nantes, France

**What is the clinical question being addressed?**

We sought to better define the incidence and causes of death of patients with ccTGA.

**What is the main finding?**

The overall incidence of mortality in ccTGA patients was 3.4 per 100 person-years, with over two-thirds of deaths due to heart failure or sudden death.

Congenitally corrected transposition of the great arteries (ccTGA) is characterized by atrioventricular (AV) and ventriculoarterial discordance^1^ with an estimated incidence of 1 per 33,000 live births.^2^ Due to the rarity of this form of congenital heart disease, there are limited data on the incidence and causes of death. To address this issue, we conducted an international multicenter retrospective study that included patients with a diagnosis of ccTGA from January 1991 to December 2018 in 29 centers across 6 countries.^3^ Institutional Review Board approval was obtained from all participating institutions. Patients were identified from local databases and medical records at each institution. All deceased and living patients diagnosed with ccTGA were eligible for inclusion. Cardiac phenotype and associated anatomic malformations were coded using the International Pediatric and Congenital Cardiac Code.^3^ Heart failure was defined as chronic diuretic use or as NYHA functional class III or IV symptoms, independent of medical treatment. Sudden death was defined as an unexpected fatal event occurring within 1 hour from the onset of symptoms, in an apparently healthy subject, or in one whose disease was not so severe to predict such an abrupt outcome. Ischemic stroke was defined as clinical evidence of cerebral focal ischemic injury based on symptoms persisting 24 hours or longer or until death, with or without cerebral imaging, and other etiologies excluded. Univariable and multivariable Cox regression models were used to assess associated factors of death. Variables associated with *P* values <0.20 in univariable analyses were included in multivariable backward selection models.

A total of 1,131 patients, 471 (41.6%) females, were enrolled. The median age at diagnosis was 0.1 years (IQR: 1 day-12.3 years). The main associated cardiac malformations were ventricular septal defect (712, 63.0%), pulmonary stenosis (476, 42.1%), and AV valve anomaly (259, 22.9%); 99 (8.7%) had a heterotaxy syndrome. Of the 1,131 patients, 419 (37.0%) underwent no repair, while 363 (32.1%) had a physiological repair, 170 (15.0%) had an anatomical repair (double switch in 139 [12.3%]; Nikaidoh, Réparation à l’Étage Ventriculaire [REV] or Rastelli in 31 [278%]), and 179 (15.8%) had a single ventricle palliation; 215 (19.0%). A permanent pacemaker was implanted in 221 (19.5%) patients.

During a median follow-up of 9.0 (IQR: 4.0-17.2) years, 105 (9.3%) patients died, 39 (37.1%) females, at a median age of 17 (IQR: 2-53) years. The corresponding incidence mortality rate was 3.4 cases per 100 person-years. Causes of death are summarized in Figure 1. Heart failure accounted for 53 (50.5%) deaths followed by sudden death (n = 18; 17.1%), postoperative complications (n = 4; 3.8%), stroke (N = 4; 3.8%), and sepsis (n = 2, 1.9%). The remaining 24 (22.9%) patients had an unknown cause of death, of whom 8 (33.3%) were aged 70 years or over. In a multivariable Cox regression analysis, factors independently associated with death were additional coexisting cardiac malformations (HR: 6.35; 95% CI: 3.08-13.08; *P* < 0.001) and complete AV block recorded at the time of diagnosis of ccTGA (HR: 3.72; 95% CI: 1.87-7.37; *P* < 0.001).

**Figure 1:**
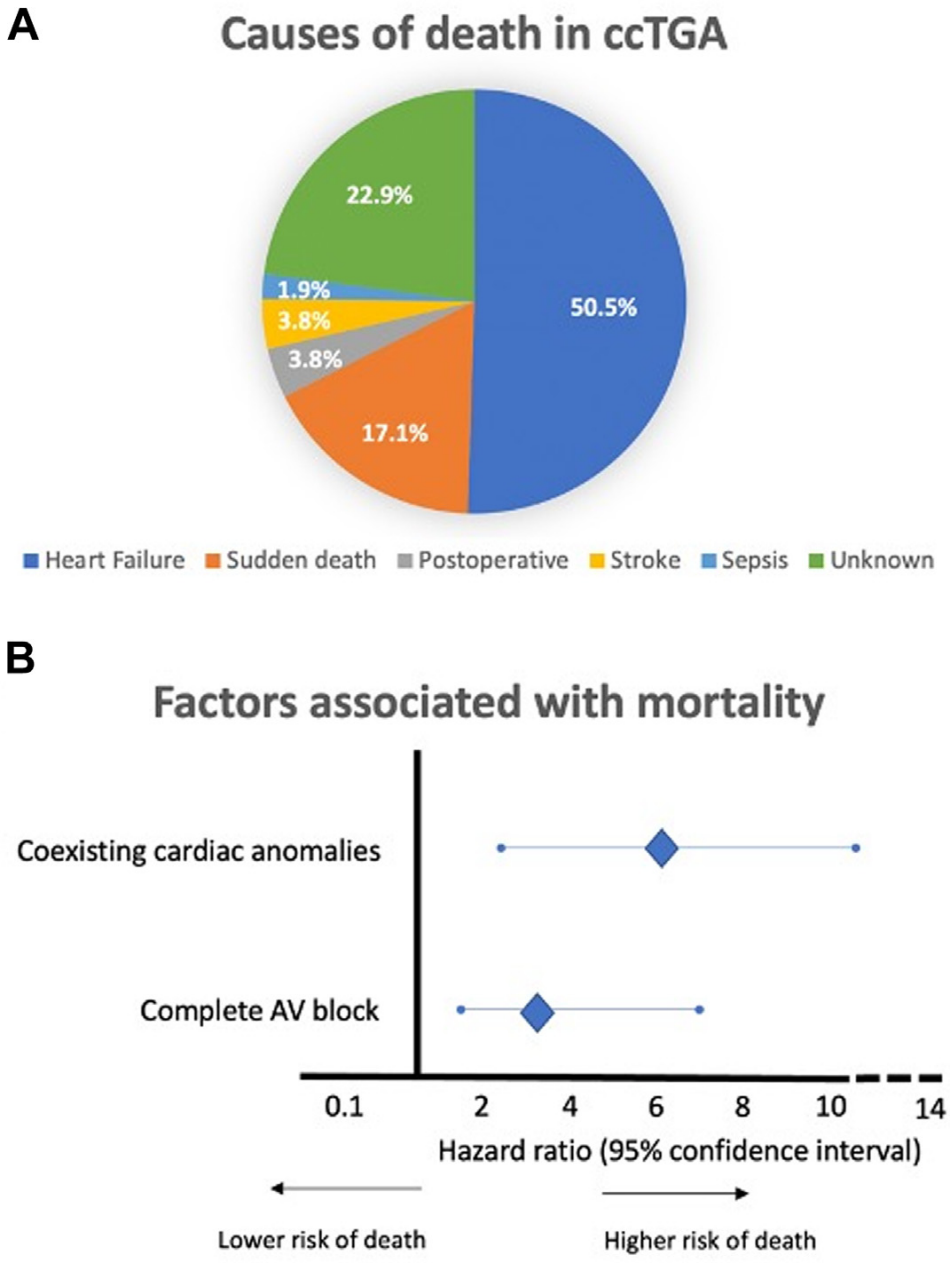
Causes of Death in Congenitally Corrected Transposition of Great Arteries Causes of death in congenitally corrected transposition of the great arteries (A); Factors independently associated with death (B). AV = atrioventricular; ccTGA = congenitally corrected transposition of the great arteries.

To our knowledge, this multicenter study is the first large-scale assessment of the incidence (3.4 cases per 100 person-years) and causes of mortality in patients with ccTGA. Heart failure deaths outnumbered all other causes of mortality combined, reflecting limitations in the ability of a morphologic right ventricle to support a systemic workload over a long-term course. No pharmacological therapy has been shown to improve survival in patients with failing systemic right ventricles and ongoing research is assessing the role of advanced heart failure therapies.^4^ The observed proportion of sudden deaths (17.1%) is higher than the 4 of 39 (10.3%) reported in a study from Australia.^5^ Disparities may reflect differences in sample sizes. Moreover, the proportion of sudden deaths is likely underestimated in our study given that causes of death were unknown in 24 (23%) patients. Consistent with the observation that additional cardiac defects are associated with higher mortality, several prior studies reported less favorable hemodynamics and greater morbidities in patients with nonisolated forms of ccTGA. The use of multiparametric and artificial intelligence-based tools could enable the development of mortality prognostic scores. Finally, the association between complete AV block and higher mortality merits further investigation. Potential pathophysiological mechanisms include loss of AV synchrony, ineffective ventricular filling, reduced cardiac output, increased systemic AV valve regurgitation, and/or pacing-induced ventricular dyssynchrony and dysfunction. Further studies are needed to determine whether more physiological pacing systems, such as cardiac resynchronization therapy, leadless pacemaker or even bundle branch pacing, can have an impact on mortality.

## References

[1] Steding G., Seidl W. (1981). Contribution to the development of the heart, part II: morphogenesis of congenital heart disease. Thorac Cardiovasc Surg.

[2] van der Linde D., Konings E.E.M., Slager M.A. (2011). Birth prevalence of congenital heart disease worldwide a systematic review and meta-analysis. J Am Coll Cardiol.

[3] Tortigue M., Nield L.E., Karakachoff M. (2022). Familial recurrence patterns in congenitally corrected transposition of the great arteries: an international study. Circ Genomic Precis Med.

[4] Fuller S. (2022). Comparing long-term sequelae of the systemic right ventricle: an overview of single versus biventricular arrangements. Semin Thorac Cardiovasc Surg Pediatr Card Surg Annu.

[5] Mccombe A., Touma F., Jackson D. (2016). Sudden cardiac death in adults with congenitally corrected transposition of the great arteries. Open Heart.

